# Food Access, Diet Quality, and Nutritional Status of Older Adults During COVID-19: A Scoping Review

**DOI:** 10.3389/fpubh.2021.763994

**Published:** 2021-11-30

**Authors:** Emily J. Nicklett, Kimson E. Johnson, Lisa M. Troy, Maitreyi Vartak, Ann Reiter

**Affiliations:** ^1^Department of Social Work, College for Health, Community and Policy, University of Texas at San Antonio, San Antonio, TX, United States; ^2^Department of Health Management and Policy, School of Public Health, University of Michigan, Ann Arbor, MI, United States; ^3^Department of Sociology, University of Michigan, Ann Arbor, MI, United States; ^4^School of Public Health & Health Sciences and Commonwealth Honors College, University of Massachusetts Amherst, Amherst, MA, United States; ^5^Department of Psychology, College of Liberal and Fine Arts, University of Texas at San Antonio, San Antonio, TX, United States

**Keywords:** food access, diet quality, nutritional status, food security, COVID-19, older adults

## Abstract

**Background:** COVID-19 has imposed challenges for older adults to access food, particularly in minority, lower income, and rural communities. However, the impact of COVID-19 on food access, diet quality, and nutrition of diverse older adult populations has not been systematically assessed.

**Objective:** To examine changes in food access, diet quality, and nutritional status among older adults during the COVID-19 pandemic and the potential differential impacts of the COVID-19 pandemic on these nutrition-related outcomes using the framework of the socio-ecological model.

**Methods:** An electronic search was conducted on 3 databases (PubMed, CINAHL, and Web of Science) on March 7, 2021. Original, peer-reviewed English-language studies published 10/1/2019–3/1/2021 were considered for which the mean age of participants was 50 years and older. In order to be considered, studies must have examined food access, food security, or nutrition constructs as an outcome.

**Results:** The initial search yielded 13,628 results, of which 9,145 were duplicates. Of the remaining 4,483 articles, 13 articles were in scope and therefore selected in the final analysis, which can be characterized as descriptive (*n* = 5), analytical (*n* = 6), and correlational (*n* = 2). Studies were conducted among community-dwelling older adult populations (*n* = 7) as well as those temporarily residing in hospital settings (*n* = 6) in 10 countries. None of the in-scope studies examined the impact of food programs or specific public policies or disaggregated data by race/ethnicity.

**Conclusions:** More research is needed to examine the impact of COVID-19 on food access/security and the differential barriers experienced by older adult populations.

## Introduction

The direct impacts of COVID-19 on the health and well-being of older adults—in terms of morbidity, mortality, and social exclusion—has received worldwide recognition in academic research, news media coverage, and increasingly, policy action. However, the indirect impact of COVID-19 on the health of older adults through food access, diet quality, and nutrition has received relatively little attention, despite the strong impact of diet quality on the health and longevity of older adult populations (1–3). These constraints are more likely to affect minority, lower-income, and rural older adult populations (4). However, the impact of COVID-19 on food access, diet quality, and nutrition of diverse older adult populations has not been systematically assessed.

Prior studies suggest that the impact of COVID-19 on diet quality among adults, in general, has been somewhat mixed. Early data from the United States Department of Agriculture (USDA) and the U.S. Census Bureau suggests there has been an increase in very low food security in the U.S., characterized when some household members reduced their food intake due to limited access to food, from 4.3% in 2018 to 9.7% in June 2020 (5–8). Other studies of adults in the U.S. have found an increase in the consumption of unhealthy foods such as heavily processed foods (9, 10) and sweets and salty snacks (9–11). Cross-national studies suggest substantial heterogeneity within and between countries in dietary changes during the COVID-19 pandemic (12), with a trend toward more unhealthy consumption during confinement (13). Among those studies citing differential impacts, diet quality has been found to vary according to socioeconomic factors (14), access to food (9, 15), and age (16). COVID-19 has been associated with dietary improvement for younger adults but negatively impacts children and older adults (16).

Older adults, as a group, are particularly vulnerable to nutritional, dietary, and food access-related disruptions as a result of COVID-19 compared to younger and middle-aged adults (16, 17). Older adults are at heightened risk of experiencing food insecurity, nutritional inadequacy, and immunosenescence (18, 19), and COVID-19 is likely to have exacerbated these problems (20). Malnutrition and poor diet quality likely affect susceptibility to, and prognosis of, COVID-19 among older adult populations (21–28). Schrack et al. (29) argue that nutritional challenges imposed by COVID-19 on older adult populations could be attributable to multiple factors: fear of going out, unavailability of healthy foods, greater consumption of processed and non-perishable foods. These challenging factors impact weight gain and weight loss with potentially detrimental effects on physical and cognitive functioning for years to come.

The purpose of this review is to characterize the peer-reviewed literature examining: (1) Changes in food access, diet quality, and nutritional status among older adults during the COVID-19 pandemic; and (2) Differential impacts of the COVID-19 pandemic on food access, diet quality, and nutrition. In addition, gaps in the literature and recommendations for future studies are identified.

The Social-Ecological Model (SEM) provides a framework to identify and describe influential factors contributing to the complexities and interdependencies between social, economic, cultural, environmental, and organizational determinants of food access (30). Multiple factors impact the availability and prioritization of food programs' response to the need for food accessibility for older adults during the COVID-19 pandemic. Understanding the landscape of an individual's food environment is an important dimension that can aid or impede an individual's ability to acquire an adequate food supply (31). The application of SEM offers a framework that considers the three “spheres of influence” to describe and evaluate factors that influence food access, diet quality, and nutritional status among older adults: intrapersonal factors (individual access), interpersonal factors (informal assistance and connections with other people), and environmental factors (organizational, community and social structures, program availability, and policies to increase access) [(32), p. 32; (30, 33)]. Examining these spheres of influence through the SEM lens can inform public health and policy implications for interventions and prevention programs that serve older adult populations during and after the COVID-19 pandemic.

The SEM model explores food insecurity by examining the effect of intrapersonal, interpersonal, and environmental factors on older adults' ability to access and sustain the resources needed to maintain proper nutrition. At the individual level, older adult's intrapersonal access to food assistance opportunities can either be hindered by their financial resources to purchase food or the presence of physical or mental health challenges that make seeking help more complicated. Interpersonal access at the community level can facilitate informal or formal assistance linking older adults with social workers and other resource navigators to gain nutritional assessments, nutritional counseling, and access to food programs (34). Finally, environmental factors at the societal level are informed by research that influences organizational, community, and social structure, program availability, and policies to increase food access to older adults (20, 34, 35).

## Methods

This scoping review summarizes current research in diet, nutritional status, food access, and food security among older adults during the COVID-19 pandemic. The purpose of this scoping review is to summarize the state of scientific research in this area and identify research gaps (36, 37). Studies were therefore not excluded due to sample size or study design type, or quality. A broad set of studies were identified and reviewed to help ensure that all relevant studies were captured.

We searched CINAHL, PubMed, and Web of Science databases to identify studies that examined nutrition, food access, food security, and diet of older adults during the COVID-19 pandemic. The key search terms we used for capturing food access during COVID-19 included food access (or food security, food insecurity, diet, nutrition), older adults (or older adult, elder, elderly), and COVID-19 (or coronavirus). Studies had to meet the following criteria to be included in this review: peer-reviewed and published articles, written in English, published between 10/1/2019 and 3/1/2021. The articles had to include data analysis at the individual level (excluding previous reviews, editorials, and commentaries). Articles also had to include nutrition, food access, food security, and/or diet as the dependent variable in analyses (or emerge in key themes in the case of qualitative analysis). Finally, the mean age of participants had to be age 50 years and older to be included. Because our sample was not restricted to industrialized societies, chose to employ a threshold of age 50 and older so that studies in differing regions and with diverse populations would be included.

All eligible studies were reviewed by two authors (MV and KJ, EN, AR, or LT). Data were abstracted on the study characteristics (i.e., specific aims, setting and sample, design, measures/outcomes, and key findings) and spheres of influence (i.e., intrapersonal factors, interpersonal factors, and environmental factors). Interpretation of study characteristics was consistent between participating authors; any differences in interpretation between the reviewers were resolved through discussion before study findings were summarized. This review provides narrative descriptions of eligible studies. In addition, this review incorporates a social-ecological model to categorize the interplay between different internal and external factors that can influence older adults' dietary and nutritional health.

## Results

**Figure 1:**
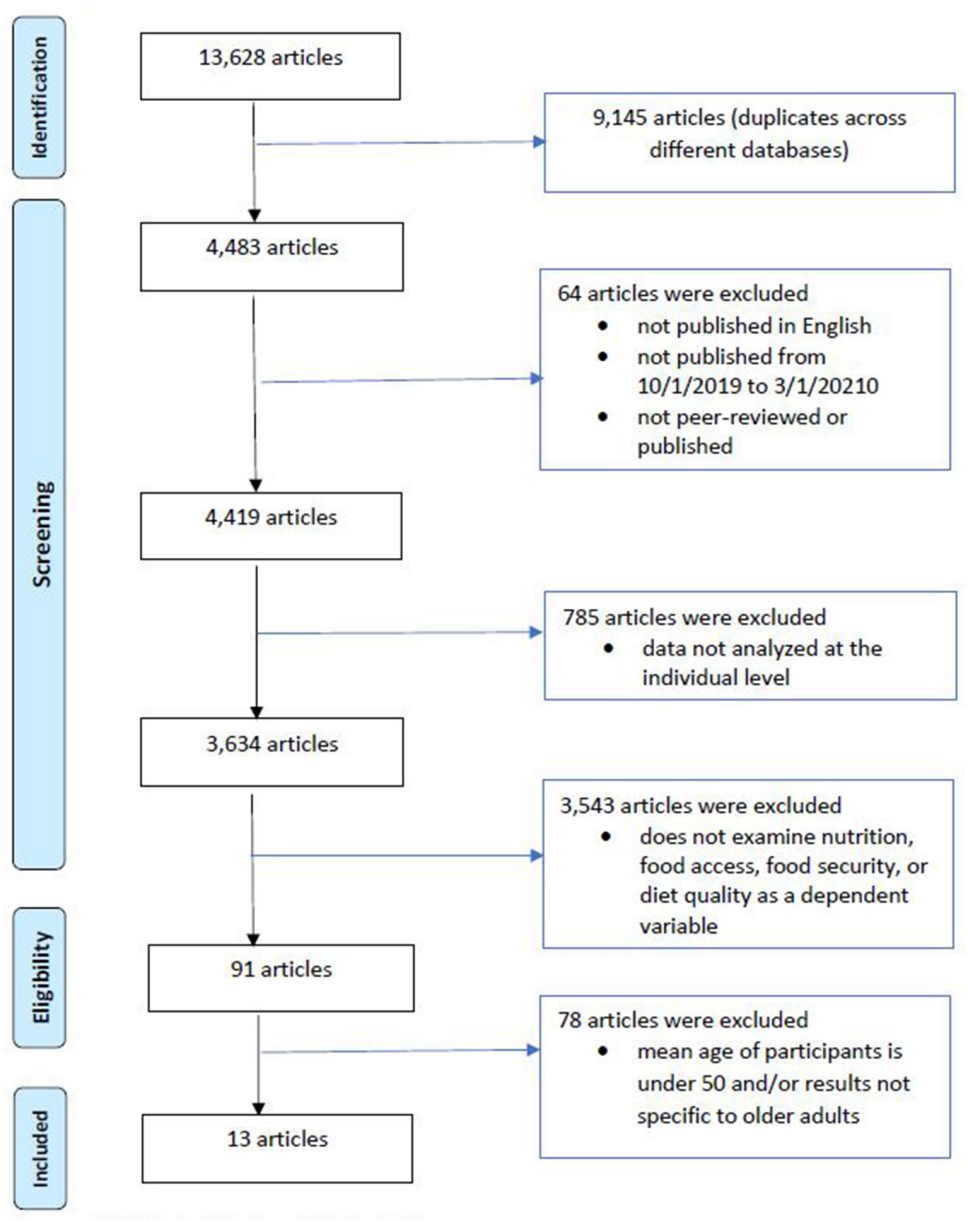
Literature search strategy: sources and exclusion criteria (published October 1, 2019 to March 1, 2021). CINAHL, PubMed, and Web of Science. Key search terms for capturing food access during COVID-19 included food access (or food security, food insecurity, diet, nutrition), older adults (or older adult, elder, elderly), and COVID-19 (or coronavirus). Above figure adapted from Moher et al. (38).

The search strategy, key terms, abstraction process, and eligibility criteria are described in Figure 1 above. Our initial search across 3 databases yielded 13,628 results^1^, of which 9,145 were duplicates. Of the remaining 4,483 articles, 13 eligible articles were included in this review: 4,470 articles were excluded because they were not published in English (*n* = 15), they were published outside of the specified dates (*n* = 31), were not peer-reviewed or published (*n* = 18), did not analyze data at the individual level (*n* = 785), did not examine nutrition, food access, food security, and/or diet as a dependent variable in the analysis (*n* = 3,543), or the mean age of participants was below the age of 50 years and/or they did not have results specific to older adults (*n* = 78).

The characteristics of the 13 studies are presented in Table 1. The studies were geographically diverse and included two studies conducted in China, Italy, and Japan. Additional studies were conducted in France, The Netherlands, Poland, South Korea, Spain, Uganda, and the United States. The studies were conducted among community-dwelling samples (*n* = 7) and those temporarily residing in hospitals (*n* = 6). Most studies used a convenience sampling approach (*n* = 12), while one study used a population-based approach. All studies were non-experimental and none of the studies involved interventions or the evaluation of programs or policies. Most studies (*n* = 10) examined cross-sectional data—two studies examined longitudinal data and one examined retrospective data. Data were quantitative in most cases (*n* = 12) with one study examining qualitative data. Two studies were web-based and 11 were conducted face-to-face, by telephone, or by mail. Sample sizes ranged from 30 to 3,219 participants, and mean ages ranged from 51.5 to 80 years.

**Table 1:** Characteristics of studies examining food access, diet quality, and nutrition among older adults during COVID-19.

**References**	**Specific aims**	**Setting and sample**	**Design**	**Measures**	**Key findings**	**Sphere of influence**
1) Bedock et al. (39)	Assess malnutrition in hospitalized patients with COVID-19, investigate links between malnutrition and disease severity at admission, study impact of malnutrition on clinical outcomes	Medicine ward at a university hospital in Paris, France *N* = 114 *M* age: 59.9	Cross-sectional, non-experimental design	Nutritional status was defined using Global Leadership Initiative on Malnutrition (GLIM) criteria	The overall prevalence of malnutrition was 42.1%, reaching 66.7% among patients admitted from ICU. No significant association was found between nutritional status and clinical signs of COVID-19. Lower albumin levels were associated with a higher risk of transfer to ICU in adjusted models	Intrapersonal
2) Cicero et al. (40)	Evaluate the effect of COVID-related quarantine on smoking and dietary habits	Population-based sample representative of Brisighella, a rural North-Italian village *N* = 359 *M* age: 64.6	Longitudinal, non-experimental design	Dietary habits were assessed using the Dietary Quality Index (DQI), a validated tool providing information on the usual food intake of 18 food items, grouped in three food categories	COVID-19-related quarantine might worsen the overall quality of the diet, leading to an increased intake of almost all categories of food. Although trends are mixed, the overall results show a trend toward decreasing diet quality that could flag future health problems	Environmental
3) Giebel et al. (41)	Explore the impact of COVID-19 public health restrictions on the lives of older adults	Convenience sample of older adults in Uganda *N* = 30 *M* age: Not reported (all age 60+)	Qualitative semi-structured interview study; non-experimental design	Diet and food access emerged in the following themes: economic impacts; lack of access to basic necessities; social impacts; and violent reinforcements of public health restrictions	Participants reported reducing food intake, in some cases to one meal a day, due to the economic impact of COVID-19 Pervasive difficulty accessing food was reported by participants, including those who had previously relied on governmental food support. Public health measures made it impossible for children to bring parents food. Violent reinforcements of public health restrictions prevented constrained opportunities to access food and other goods	Environmental
4) Górnicka et al. (42)	Identify patterns of dietary changes during COVID-19 pandemic	Polish residents aged 18 and older*N* = 2,381*M* age: Not reported (age groups included 50–59 and 60+)	Rapid, large cross-sectional online survey, non-experimental design	Questionnaire assessed “Impact of the COVID-19 Pandemic on the Diet and Lifestyle of adults” (PLifeCOVID-19), which measured patterns associated with dietary change, including Prohealthy, Constant, and Unhealthy	Older adults were significantly less likely to follow the Prohealthy pattern compared to those aged 30 and younger (67% lower in respondents aged 50-59 years and 78% lower in respondents aged 60+. Older adults were significantly more likely to follow the Constant pattern compared to those aged 30 and younger (3 × higher in respondents aged 50-59 and 2.8 × higher in respondents aged 60+). Adherence to the Unhealthy pattern was not significantly associated with age group	Intrapersonal/ Environmental
5) Im et al. (43)	Determine the nutritional status of COVID-19 patients, particularly as it pertains to immunity	Adults with COVID-19 admitted to Inha University Hospital, South Korea *N* = 50 *Median* age: 57.5	Cross-sectional, non-experimental design using a control group for 25-hydroyvitamin D3	Nutrient levels assessed included vitamin B1, B6, B12, vitamin D (25-hydroyvitamin D3), folate, selenium, and zinc	Severe vitamin D deficiency was found in 24% of patients in the COVID-19 group and 7.3% in the control group. A deficiency of vitamin D or selenium may decrease the immune defenses against COVID-19 and cause progression to severe disease	Intrapersonal
6) Li et al. (22)	Evaluate the prevalence of malnutrition and its related factors in older patients	COVID-19 patients admitted to Wuhan Tongji Hospital, China *N* = 182 *M* age: 68.5	Cross-sectional, non-experimental design	Nutritional status was assessed using the Mini Nutritional Assessment (MNA). Participants were categorized into non-malnutrition (MNA ≥ 24), risk of malnutrition (MNA 17–23.5) and malnutrition groups (MNA score <17)	The prevalence of malnutrition was high: 27.5% were in the group with malnutrition risk and 52.7% were in the malnutrition group. Nutrition support should be strengthened during treatment, especially among those with diabetes mellitus, low calf circumference, or low albumin	Intrapersonal
7) Niles et al. (44)	Assess food insecurity, food access, coping strategies, and suggested potential interventions among food secure, consistently food insecure, and newly food insecure respondents	Convenience sample using Limesurvey in Vermont, USA *N* = 3,219 *M* age: 51.5	Cross-sectional, non-experimental design	United States Department of Agriculture six-item validated food security module to measure food insecurity before and since COVID-19	Overall, there was a nearly one-third increase in household food insecurity, with 35.5% of food insecure households classified as newly food insecure. Two-thirds of food insecure households eat less since COVID-19. Age was not significantly associated with food insecurity in this study	Interpersonal/ Environmental
8) Otaki et al. (45)	Examine the relationship between dietary variety and frailty during COVID-19 restrictions on outings	The study was conducted among Japanese women *N* = 322 *M* age: 80.0	Cross-sectional, non-experimental design	A dietary variety score (ranging from 0 to 10 points) was used to assess the food group intake	Diet was correlated with frailty in older adults living in the community during the period of restriction on outings due to COVID-19	Intrapersonal/ Environmental
9) Pironi et al. (46)	Evaluate the prevalence of malnutrition and provided nutritional therapy	Clinical audit on adult patients hospitalized for COVID-19 in Bologna, Italy *N* = 268 *Median* age: 74	One-day clinical audit of nutritional status and nutritional therapy	Assess malnutrition risk and diagnosis of malnutrition using modified Nutritional Risk Screening 2002 tool (NRS-2002) and modified Global Leadership Initiative on Malnutrition (GLIM) criteria	Most patients were at nutritional risk and one-half of them were malnourished. The frequency of nutritional risk, malnutrition, and decreased hospital diet intake differed by intensity group setting. Patient energy and protein intakes were at the lowest limit or below the recommended amounts, indicating the need for actions to improve nutritional care practice	Environmental
10) Ruiz-Roso et al. (47)	Examine the impact of the COVID-19 lockdown on nutrition and exercise habits	Patients with type 2 diabetes from the University Hospital La Princesa in Madrid, Spain *N* = 72 *M* age: 63	Cross-sectional, non-experimental design including network mapping and analyses	A food frequency questionnaire (FFQ), food craving questionnaire-state (FCQ-S) and food craving questionnaire-trait (FCQ-T) were used	Increases in vegetable, sugary food, and snack consumption were found. An association between levels of food cravings and snack consumption was also found	Intrapersonal/ Environmental
11) Shinohara et al. (48)	Clarify association between frailty and changes in lifestyle	Community-dwelling older adults residing in Takasaki City, Japan, helped regularly by volunteers *N* = 856 *M* age: 78.4	Cross-sectional, non-experimental design	As part of the Questionnaire for Change of Life (QCL), participants were asked about changes in meal size in the past 6 months during the pandemic	Meal size decreased significantly among older adults with frailty (compared to those without frailty) during the COVID-19 pandemic in Japan	Intrapersonal/ Environmental
12) Visser et al. (49)	Examine the self-reported impact of COVID-19 pandemic on nutrition and physical activity behaviors in Dutch older adults	Longitudinal cohort study of community-dwelling older adults in Amsterdam, the Netherlands *N* = 1,119 *M* age: 74	Longitudinal, non-experimental design	Frequency of perceived changes in nutrition behaviors during the past weeks due to COVID-19 (difficulty obtaining groceries, skipping warm meals, eating less than normal, eating too little or losing weight, and snacking more	An impact on nutritional behavior predisposing to overnutrition (e.g., snacking more) was reported by 20-32% and undernutrition (e.g., skipping warm meals) was reported by 7–15% of participants. COVID-19 appears to have a negative impact on nutrition and physical activity behavior of many older adults	Intrapersonal/ Environmental
13) Zhao et al. (26)	Identify nutritional risk and examine association with mortality risk among COVID-19 patients	West Campus of Union Hospital in Wuhan, China*N* = 413*M* age: 60.31	Retrospective, observational study	Nutritional risk was assessed using Nutritional Risk Screening 2002 (NRS), including nutritional status (based on weight loss, BMI, and food intake) and disease severity. An NRS total score of >3 was considered “at risk.” Other nutritional biomarkers were assessed	Most patients, especially critically ill patients, had significant changes in nutrition-related parameters. Critically ill patients had significantly higher NRS scores, which were correlated with nutrition-related markers. Only 24% of at risk patients received nutritional support. Most severely and critically ill patients with COVID-19 are at nutritional risk. Patients with higher nutrition risk had worse outcomes and required nutrition therapy	Intrapersonal/ Environmental

*Intrapersonal factors refer to factors that impact individual access to resources (e.g., demographics, financial, mental health, physical health, and functional status). Interpersonal factors refer to factors that require informal assistance or connections with other people (e.g., emotional and financial support). Environmental factors refer to factors caused by related to an individual's surrounding (e.g., institutional, community and social structures, program availability, and policies to increase access)*.

### Key Measures

Twelve of the thirteen in-scope studies examined diet, nutritional status, food access, and/or food security as a dependent variable. The specific dependent variables varied widely across studies and can be generally grouped as (1) *Nutritional status, malnutrition, and nutrient levels*; (2) *Food security and access;* (3) *Dietary habits, dietary variety, and food group intake;* (4) *Meal size and meal frequency; and* (5) *Food cravings*. Many of the key measures are directly linked to diet quality. Food security and access along with meal size and frequency impact the quantity and quality of food intake. Diet habits, variety, and food group intakes capture aspects of an overall healthful or less healthful diet. In addition, one study qualitatively examined the impact of COVID-19 on older adults, and relevant qualitative themes emerged.

Measures of *nutritional status, malnutrition, and nutrient levels* included the Global Leadership Initiative on Malnutrition (GLIM) criteria (39, 46), the Mini Nutritional Assessment (MNA) to determine malnutrition (22), the Nutritional Risk Screening 2002 Tool (NRS-2002) for malnutrition risk (26, 46), and the assessment of nutrient levels, including vitamin B1, B6, B12, D, as well as folate, selenium, and zinc from blood samples (43). Measures of *food security and access* included measures of reported difficulty obtaining groceries (49) and the USDA six-item short form validated food security module to measure food security before and since COVID-19 (44). Measures of *dietary habits, dietary variety, and food group intake* included the Dietary Quality Index (DQI) for dietary habits (40), patterns of dietary change (42), dietary variety (45), and compliance with dietary guidelines and recommendations (47). Diet in these studies were measured by the DQI Questionnaire (40), specific questions on food group changes (42), a questionnaire on dietary intake and habits (45), and a Food Frequency Questionnaire [FFQ; (47)].

Measures of *meal size and meal frequency* included the Questionnaire for Change of Life (QCL). This survey measured changes in meal size in the past 6 months (48) and the frequency of perceived changes in skipping warm meals, eating less than normal, eating too little or losing weight, and snacking more (49). Measures of *food cravings* included the Food Craving Questionnaire-State (FCQ-S) and the Food Craving Questionnaire-Trait (FCT-T) (47). In the qualitative study by Giebel et al. (41), the authors used a qualitative semi-structured phone-based interview to capture the impact of COVID-19 on the lifestyle of older adults including changes in diet and access to food.

### Study Aims and Key Findings

The aims of the thirteen studies can be characterized as descriptive, analytical, and correlational. First, the descriptive studies (*n* = 5) assessed malnutrition among hospitalized patients with COVID-19 (22, 39), investigated linkages between malnutrition and disease severity at admission (39, 46), and studied the impact of malnutrition on clinical outcomes of COVID-19 among older adults (26, 39), including links to immunity (43). These descriptive studies reported the prevalence of malnutrition among hospitalized patients to be 42.1 to 52.7% (22, 39, 46), with higher rates of malnutrition among critically ill patients (26, 39, 46). Of note, one study reported no significant association between nutritional status and clinical signs of COVID-19 (39). Additional risk factors for malnutrition included lower albumin levels (22, 39), low calf circumference (22), and diabetes mellitus comorbidity (22). Vitamin D or selenium deficiency was also common among COVID-19 patients (24 vs. 7.3% among controls), which weakens immune system defenses against initial infection—and the progression of—COVID-19 (43).

Second, the analytical studies (*n* = 6) examined the impact of COVID-19 on the lives of older adults, including nutrition and dietary habits (40, 42, 47, 49) and food security, food access, coping strategies, and suggested potential interventions (44). In addition, one study (41) collected qualitative data on the impact of COVID-19 public health restrictions on the lives of older adults. Across and within studies, the findings on the impact of COVID-19 on eating patterns and diet quality among older adults were mixed. While one study found that older adults were more likely than younger adults to maintain their dietary patterns (42), other studies found an increase in intake of almost all categories of food (40), including vegetable, sugary food, and snack consumption (47, 49). One other study of older adults in the United States (44) found nearly a one-third increase in household food insecurity since COVID-19 among participants, with 35.5% of food insecure households classified as newly food insecure (44). Findings from quantitative (44) and qualitative (41) studies documented physical barriers, economic barriers, and challenges pertaining to food access during COVID-19. Reductions in food intake were found in several studies (41, 44, 49), including as many as two-thirds of households who experienced household food insecurities eating less since COVID-19 (44). In the qualitative study of older adults in Uganda (41), diet and food access emerged in themes related to economic impacts, lack of access to basic necessities, social impacts, and violent reinforcements of public health restrictions.

Third, the correlational studies (*n* = 2) examined the relationship between dietary variety and frailty (45) and between meal size and frailty (48) during COVID-19 restrictions and stay-at-home orders. In these studies, less dietary variety (45) and smaller meal size (48) were significantly positively associated with frailty among community-dwelling older adults during the COVID-19 outing restrictions.

### The Social Ecological Model and Spheres of Influence

Using the SEM approach, the in-scope articles included singular (e.g., intrapersonal and environmental) and hybrid spheres of influence (e.g., intrapersonal/environmental and interpersonal/environmental), as shown in Table 1. Three articles specifically focused on intrapersonal factors. These factors include the presence of nutritional deficiencies, prevalence, and severity of malnutrition among hospitalized patients, leading to adverse reactions to normal human functioning and affecting specific clinical outcomes (22, 39, 43). Three articles discussed the singular sphere of environmental influence at the institutional, policy, community, and social structure levels. Both varying levels of government-enforced quarantine and public health restrictive measures (e.g., social distancing and curfew) to reduce the risk of COVID-19 were found to limit physical activity and increased sedentary behavior that led to changes in food consumption (40, 41). In the clinical setting, nutritional assessments found that patients hospitalized for COVID-19 had a high prevalence of nutritional risk and malnutrition (46). Community and social structures may alter an individual's access to food resources, contributing to malnutrition before hospitalization for COVID-19.

This scoping review identified two hybrid spheres of influence. Six articles focused on the intrapersonal/environmental spheres of influence, specifically an individual's access to resources and response to COVID-19 lockdown restrictions or institutional practices that can affect nutrition, access to nutritional therapy, and potential health consequences among community-dwelling and hospitalized adults (26, 42, 45, 47–49). Only one article involved the interpersonal/environmental spheres of influence, specifically how a statewide stay-at-home orders, policies and COVID-19 impacted food insecurity and disrupted food access; the latter was associated with many adverse individual and public health outcomes (44).

## Discussion

The findings from reviewed studies point to the importance of understanding the impact of COVID-19 on food access, diet quality, and nutritional status of older adult populations. While some studies found that food access, diet quality, and nutritional status were maintained or even improved among older adults during COVID-19, the majority of studies found challenges in these areas for older adults. The in-scope studies pointed to differences in nutritional risk during COVID-19 among older adults, with higher risk of food insecurity, challenges to food access, and/or poorer diet quality among those who experienced financial insecurity, job loss/disruptions, and among those who experienced functional limitations, frailty, or were underweight. Despite a wide geographic diversity of study settings, a notable omission of the in-scope studies is the examination of differences in food security, food access, and diet quality by race/ethnicity. Another notable gap in the examined studies is the examination of the impact of food programs or specific public policies on food access, diet quality, and nutritional status of diverse older adult populations. Further research on the impact of COVID-19 on food security, access, and diet quality among diverse older adult populations is needed, as is research on the effectiveness of interventions and policy strategies to address these unmet needs.

Malnutrition/nutritional deficiency appears to be linked to both susceptibilities to COVID-19 and the severity of COVID-19 outcomes in older adults. In the descriptive studies examined here, hospitalized patients with COVID-19 were found to be at greater risk of experiencing malnutrition and lacking essential micronutrients (22, 39). Patients experiencing malnutrition at admission were also more likely to have worse COVID-19 related outcomes (46). Although one study did not support this finding (39), the linkages between (a) malnutrition and susceptibility to COVID-19 and (b) malnutrition and severity of COVID-19 outcomes are further supported by the higher rates of malnutrition found among critically ill patients in examined descriptive studies (26, 39, 46). Further, there is evidence that the infections, loss of appetite, and damage in the digestive system from COVID-19 could cause additional nutritional risk (26). Further discussion of these linkages is beyond the scope of this review, however, the role of nutrition in both susceptibility to COVID-19 and COVID-19 related outcomes punctuates the importance of identifying and addressing barriers to food access, food security, and diet quality among hospitalized and community-dwelling older adult populations alike through such strategies as nutritional screening among older adults with COVID-19. In the current review, two studies paired nutritional risk assessment tools with blood measures, including albumin (22, 39). The collection of blood measures associated with nutritional status increased confidence in the results by providing multiple measures of nutritional status and may be a reasonable approach in future studies. We recommend that future studies prospectively examine multiple measures of nutritional status, food access, and COVID-19 status over time among community-dwelling older adults and those in hospital/nursing home settings. The examination of the relationships between repeated measures will help disentangle the relationships between nutritional status, immunity/susceptibility to COVID-19, and outcomes/severity of COVID-19.

Findings from the analytical studies suggest that COVID-19 has differential impacts on the diet quality, food security, food access, and coping strategies among different older adult populations. While some studies found that conditions in these factors maintained or improved during COVID-19, other studies found that these were strained during COVID-19. In a study of diet quality, Górnicka et al. (42) found that among Polish adults, those aged 60 years or older were nearly three times more likely to maintain their dietary pattern compared to those 30 years or younger, which is supportive of observations in prior research regarding the consistency of dietary intake among older adults (50). In a study of diet quality among older adults in Spain with type 2 diabetes during COVID-19, Ruiz-Roso et al. (47) found that vegetable intakes increased, along with the consumption of sugary foods and snacks. Several authors have suggested that improvements in dietary intake, such as increased intakes of vegetables, whole grains, and other healthful foods, may be due to increased cooking due to increased time at home during the COVID-19 quarantine (47, 51). Previous studies show that home-prepared meals are of higher diet quality compared to away from home meals (52–54). However, the positive association between diet quality and eating at home appears to be influenced by income, with higher-income adults having a more positive association with diet quality (55). The disruption of the COVID-19 pandemic should not be underestimated and may differentially impact vulnerable populations.

Alongside the greater consumption of foods in general found in some studies [e.g., (47)], other studies reported reductions in food intake or worsening of diet quality due to COVID-19 (41, 44, 48, 49). Niles et al. (44) found that food insecurity increased by nearly 30% during the COVID-19 pandemic among older adults in predominantly rural Vermont, with over 35% of food insecure households classified as newly food insecure. The authors state that the main reason for increased food insecurity was job loss or disruption (e.g., fewer hours). Compared to food secure households, food insecure households expressed greater challenges to food availability (e.g., not finding the types of preferred foods) and food access (e.g., going to more places than usual to find food; and inability to afford foods). These findings were echoed in a qualitative study of older adults in Uganda by Giebel et al. (41), which reported participants reduced their food intake to as little as one meal a day due to the economic impact of COVID-19. While disruptions in the food and agriculture supply chain were partially to blame, the reduction in food intake had also been facilitated in several ways by public health policies meant to protect people against COVID-19, constraining opportunities for older adults to access foods or for children to bring food to their older adult parents (41).

The impact of COVID-19 on food access, diet quality, and nutritional status of older adult populations was not consistent within or across studies. Furthermore, the apparent impact of factors such as relative age and gender were not clear-cut; men experienced a greater risk of malnutrition in one study (39), while women experienced a greater risk of food insecurity in another study (44). It appeared that women were more likely than men to report changes in diet (49), including increases in snack intake during COVID-19 (47). While relatively older age was found to be associated with eating behaviors related to undernutrition such as eating less than normal and skipping meals during COVID-19 in some studies (49), relatively older adults were found to be more likely to maintain their dietary habits during COVID-19 relative to other age groups in other studies (42, 47). On the other hand, younger older adults, especially women, were more likely to have increased snacking and alcohol intake and behaviors, which may lead to overnutrition (49). This could be due to differential risk factors among study samples, but could also reflect social vulnerabilities to food insecurities more generally. The findings of the studies in our analysis draw attention to risk factors beyond age and gender that are associated with higher risk of undernutrition during COVID-19, including frailty, functional limitations, or being underweight (45, 48, 49), living alone (49), or experiencing job loss (44). Therefore, the findings from in-scope studies suggested that the pandemic may have impacted different older adult age groups in different ways and may be useful in developing and informing ongoing interventions to target specific populations at risk of food insecurity or nutritional risk.

One strength of the included studies is the geographic diversity of samples represented—including samples from Asia, Europe, Africa, and North America. In-scope studies included older adult populations hospitalized for COVID-19 as well as community-dwelling older adults. However, one limitation is the reliance on convenience samples and cross-sectional data: of the thirteen in-scope studies, only two were part of an ongoing longitudinal study (40, 49), enabling the examination of repeated measures of dietary outcomes. Twelve of the thirteen studies used convenience samples, often relying on clinical populations or those participating in web-based surveys, limiting the extent to which the findings can be generalized to diverse older adult populations. Notably, none of the in-scope studies examined differences in food security, food access, or diet quality by race/ethnicity. Differential access to healthy food options by race/ethnicity has long been acknowledged in the United States and other societies (56, 57).

Incorporating an SEM approach provides an important perspective to examine food insecurity as an interconnected process that involves understanding the structural contexts that can have short-term and long-term impacts on older adults' health and well-being. The linkage between these different spheres of influence at the intrapersonal, interpersonal, and environmental levels is complex. However, it is necessary to gain deeper insight into how individuals' interactions with varying spheres of influence are affected by stay-at-home policies to safeguard residents during the COVID-19 pandemic (26, 42, 45, 47–49). It is vital that future research evaluate the efficacy of COVID-19 multilevel interventions that address food insecurity and downstream public health impacts through food-support programs, screening measures, nutritional, and behavioral counseling (20, 35) on older adult populations.

The COVID-19 pandemic has further exposed the systemic health and social inequities throughout the United States and other countries. These inequities have led to an influx of conversations about the importance of advocating for research, interventions, and actionable policies that advance health equity and address the unequal impacts of this pandemic on older adults' health and well-being. Further examination of how COVID-related challenges to food security, access, and diet quality differ between racial/ethnic majority populations and minoritized populations is critical for identifying the root causes of inequities but also addressing them during the present and future crises. While other studies have examined the differential impacts of COVID-19 on food security, food access, or diet quality by race/ethnicity, they were outside of the scope of this study because they did not report findings specific to older adult populations (58, 59). Studies have found an increased incidence of food insecurity among minoritized groups due to lower availability of healthy food choices and nutritional education, increased rates of poverty, and decreased access to quality healthcare in the U.S. (58–60). We encourage future research to examine food equity among diverse older adult populations, during COVID-19 and in future health, human, and environmental disasters.

The COVID-19 pandemic has shown that a public health response to food insecurity should identify and disentangle barriers to access. The pandemic has also shown that the public health response should not be divorced from federal and state legislation or from program administration at federal, state, and local levels (61, 62). The pandemic-related increase in food insecurity has been further complicated by social distance policies that can hinder older adults' ability to benefit from food security interventions. Effective strategies include external supports to address economic and physical barriers, such as extra money for food or bills, support for delivery costs, and information about and help with applying to food assistance programs (44). In addition to individual resources, older adults benefit from collective resources in the community providing food access during the COVID-19 pandemic and lockdown periods.

Food insecurity is a chronic, longstanding issue that has been worsened during COVID-19. Inequitable access to food programs and resources disproportionately impacts Non-Hispanic Black, Hispanic, indigenous, low-income households, and those living with chronic diseases and disabilities, further exacerbating existing disparities among the most under-resourced groups (20, 63). These differences in distribution and access can lead to health inequities impacting how older adults live and age. The strategies recommended to address food insecurity before the pandemic—such as improving public transportation, increasing availability of high quality, affordable foods in local grocers, and decreasing barriers to participation in food programs among food insecure individuals (64)—are still critical for addressing food insecurity and other barriers to access. Programs to increase food access and diet quality among diverse older adults have been effective in increasing access to fresh fruits and vegetables (65). However, due to the disparate impact of COVID-19 on specific groups, including minority older adults, low-income households, and older adults with frailty/disabilities, more work is needed to address social determinants of food access and diet quality.

## Conclusions

This study lays the foundation for further examining structural influences on diet, nutritional status, food security, and food access and evaluating policies, programs, and interventions that can improve nutrition-related outcomes for diverse older adults. While we are steadily moving toward decreased COVID-19 cases in many places, areas in most countries are witnessing a resurgence. This cycle is likely to continue for some time, and we must be better prepared for future pandemics and public health challenges. Therefore, there is a need for both continued assessment of the immediate impact of COVID-19 and the long-term health implications of barriers to food access, diet quality, and nutrition of older adults. Future research should examine effectiveness and equity in implementing interventions, programs, and policies to address these barriers in diverse older adult populations.

## Data Availability Statement

The original contributions presented in the study are included in the article/supplementary material, further inquiries can be directed to the corresponding authors.

## Author Contributions

EN, KJ, and LT contributed to the conception and study design and wrote sections of the manuscript. KJ and MV conducted the database searches. MV, KJ, EN, and AR contributed to the coding/abstraction of studies. All authors contributed to the manuscript revision, read, and approved the submitted version.

## Funding

This work was supported by a Health, Community and Policy (HCaP) Pilot Grant Award from the University of Texas at San Antonio. This was also supported by an award from the National Institute on Aging of the National Institutes of Health (award number T32AG000221) and an award from the National Institute of Food and Agriculture, U.S. Department of Agriculture, the Center for Agriculture, Food and the Environment and the Nutrition department at University of Massachusetts Amherst (project number MAS00564).

## Author Disclaimer

The content is solely the responsibility of the authors and does not necessarily represent the official views of the National Institutes of Health, U.S. Department of Agriculture, or the National Institute of Food and Agriculture.

## Conflict of Interest

The authors declare that the research was conducted in the absence of any commercial or financial relationships that could be construed as a potential conflict of interest.

## Publisher's Note

All claims expressed in this article are solely those of the authors and do not necessarily represent those of their affiliated organizations, or those of the publisher, the editors and the reviewers. Any product that may be evaluated in this article, or claim that may be made by its manufacturer, is not guaranteed or endorsed by the publisher.

## Footnotes

^1^The initial searches yielded a total of 13,628 results, of which 12,233 were from CINAHL, 1,289 were from PubMed, and 106 were from Web of Science.
